# Diffuse large B-cell lymphoma with intussusception in adults: a case report and literature review

**DOI:** 10.3389/fmed.2025.1717969

**Published:** 2026-01-05

**Authors:** Kang He, Shijie Cai, Fangfang Xie, Qicui Pan, Yanxiu Liu, Yongzhen Li, Xuan Lin

**Affiliations:** 1 Department of Emergency Surgery, First Affiliated Hospital of Gannan Medical University, Ganzhou, China; 2 Department of Clinical Laboratory, First Affiliated Hospital of Gannan Medical University, Ganzhou, China; 3 First Clinical Medical College, Gannan Medical University, Ganzhou, China

**Keywords:** intussusception, B-cell lymphoma, diffuse large B-cell lymphoma, non-Hodgkin’s lymphoma, gastrointestinal lymphoma, case report

## Abstract

Intussusception secondary to gastrointestinal diffuse large B-cell lymphoma (DLBCL) is exceedingly rare in adults. We present a case of a 23-year-old male admitted with abdominal pain as the primary symptom. Computed tomography (CT) revealed an ileocolic intussusception involving the ascending and transverse colon, demonstrating a characteristic “target.” Conservative treatment (including air enema) was ineffective, and the patient finally received surgical treatment. The resected lesion was pathologically confirmed as DLBCL. The patient received postoperative chemotherapy with the R-CHOP regimen. At the 5-month follow-up, no complications or evidence of lymph node involvement elsewhere was observed. Adult intussusception patients should be highly alert to the possibility of malignant tumors, especially lymphoma. Early diagnosis and standardized comprehensive treatment are critical for improving long-term prognosis.

## Introduction

Intussusception is one of the common acute abdominal conditions clinically defined as a pathological state in which a segment of the intestine prolapses into an adjacent intestinal lumen, leading to obstruction of intestinal contents. It frequently occurs in infants and young children, while adult intussusception is exceedingly rare, with an incidence of only 2–3 cases per million (1–3). Unlike pediatric cases, which are mostly idiopathic, approximately 70%–90% of adult intussusception cases have identifiable pathological causes, primarily including neoplasms, polyps, inflammatory conditions, and other organic bowel lesions. Among these, malignant tumors account for over 50% (4, 5). The most common culprits are primary gastrointestinal adenocarcinomas or metastatic cancers, whereas intussusception caused by lymphoma is rare. Notably, 90% of such lymphoma-related cases are B-cell non-Hodgkin lymphomas (NHL) (6–8).

Diffuse large B-cell lymphoma (DLBCL) is the most common pathological subtype of non-Hodgkin lymphoma (NHL), accounting for 30%–40% of adult NHL cases, and is classified as a highly aggressive B-cell lymphoma. DLBCL can involve any lymphatic tissue throughout the body, with gastrointestinal tract involvement occurring in approximately 5%–20% of cases (9). Intussusception is a rare but serious complication of gastrointestinal DLBCL. Clinical manifestations are often subacute or chronic recurrent symptoms (e.g., abdominal pain, diarrhea, vomiting) and lack specificity, making it easy to misdiagnose as appendicitis, intestinal obstruction, or other organic bowel diseases (10). This article reports the diagnosis and treatment process of a 23-year-old young male diagnosed with gastrointestinal DLBCL complicated by intussusception. By reviewing previous literature, we aim to enhance understanding of this disease and explore more precise diagnostic and individualized treatment strategies to further improve the management of such rare conditions.

## Case description

A 23-year-old adult Chinese male was admitted to the hospital with a chief complaint of “recurrent intermittent abdominal pain for 3 weeks, aggravated for 3 days.” The patient reported the onset of abdominal pain 3 weeks prior, which occurred intermittently and resolved after self-administered antibiotics. The pain recurred 3 days ago with worsening intensity. The patient was in fair general condition, maintained a normal diet, and reported changes in bowel habits. No fever, night sweats, or significant weight loss were observed. His past medical history was unremarkable, with no history of infectious diseases or previous hospitalizations. There was no significant family history of similar conditions. Furthermore, no family history of any other gastrointestinal malignant tumors was documented.

Physical examination revealed a flat abdomen, bowel sounds approximately eight times per minute, and no shifting dullness. Significant tenderness and rebound tenderness were noted in the right lower quadrant, without a palpable abdominal mass. The liver and spleen were not palpable.

**FIGURE 1:**
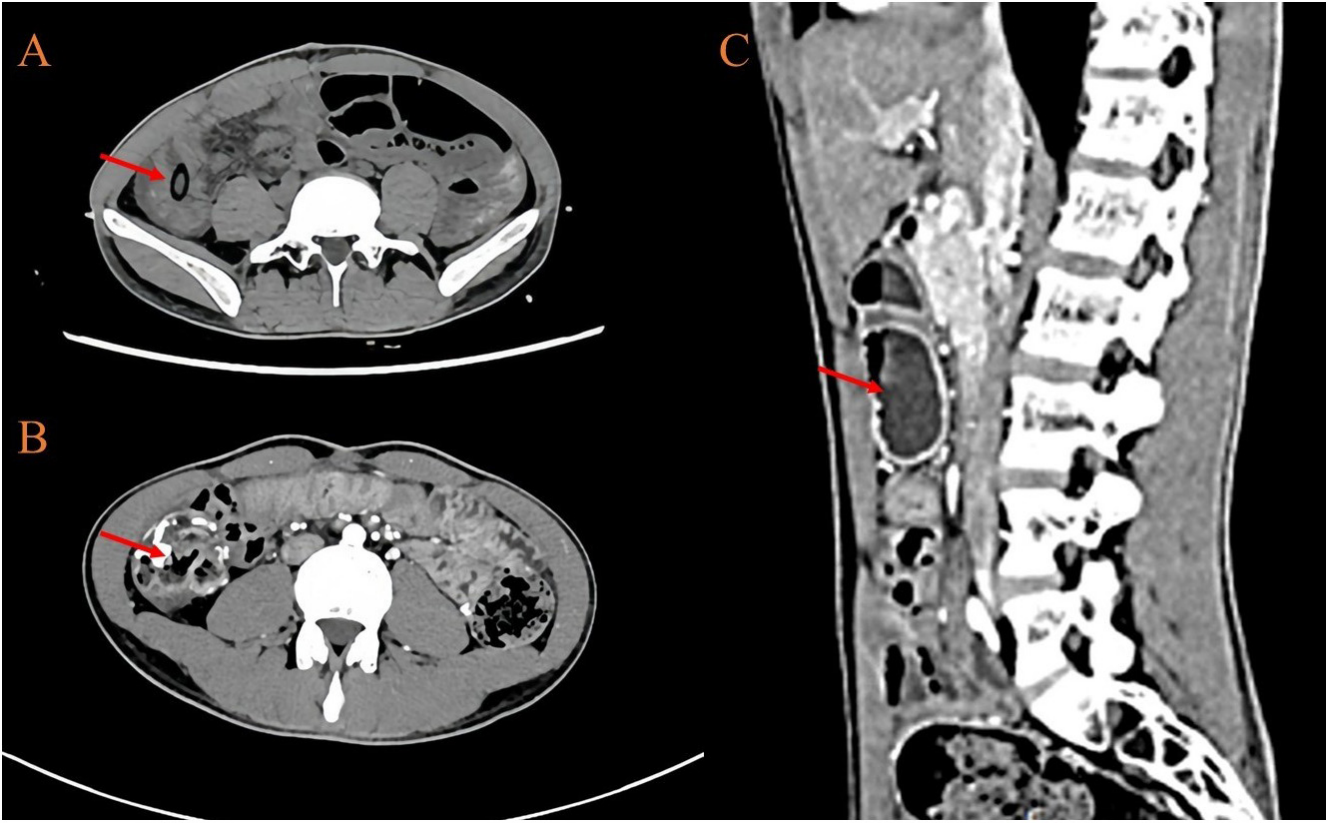
**(A)** An axial CT image where the arrow indicates the “target”; **(B)** An axial CT image where the arrow points to the ileocecal region; **(C)** A sagittal CT image where the arrow demonstrates dilated bowel loops; The findings indicate the presence of an intussusception involving the ascending colon and transverse colon, where a segment of the intestine along with its mesentery has telescoped. The local appearance of the “target” is characteristic and supports the diagnosis of intussusception.

Laboratory tests showed a hemoglobin level of 116 g/L and ferritin level of 15.8 ng/L, with no other remarkable abnormalities. Plain and contrast-enhanced computed tomography (CT) demonstrated: intussusception involving the ascending colon and transverse colon with mesentery, showing a characteristic “target” locally; a small amount of fluid in the abdominal and pelvic cavities; no other abnormal densities or enhancement patterns were observed (See Figure 1).

The contrast-enhanced abdominal CT scan showed no abnormal densities or enhancement signals. Furthermore, given the patient’s age of 23, the condition was not deemed to be caused by an intestinal tumor. The patient underwent an attempt of pneumatic reduction, which was unsuccessful. Subsequently, diagnostic laparoscopy was performed, revealing significant edema of the terminal ileum and an ileocolic intussusception. No abnormal masses were detected in the rest of the bowel. Following an unsuccessful attempt at laparoscopic reduction, and owing to the significant risk of iatrogenic bowel injury associated with further maneuvers, the procedure was converted to an open laparotomy.

A midline periumbilical incision approximately 8 cm in length was made, and the abdomen was entered in layers. After exteriorizing the ileocecal region, manual reduction of the intussuscepted bowel was performed. A firm mass measuring approximately 4 cm × 4 cm was identified in the terminal ileum. The corresponding mesentery exhibited cord-like multiple nodules extending approximately 6 cm. The distal colon showed localized firmness with circumferential wall thickening, and a mass measuring approximately 2 cm × 1 cm was palpated in the adjacent mesentery. The lesion demonstrated clearly demarcated margins. A comprehensive re-examination of the entire small intestine and colon identified no additional abnormalities. The operative impression was consistent with an intestinal neoplasm; however, a definitive characterization was not possible at that time. Therefore, resection was performed, including 15 cm of the ileum proximal and 5 cm of the colon distal to the intussusception site, encompassing all visible masses and the involved mesentery. A drainage tube was placed in the surgical area.

**FIGURE 2:**
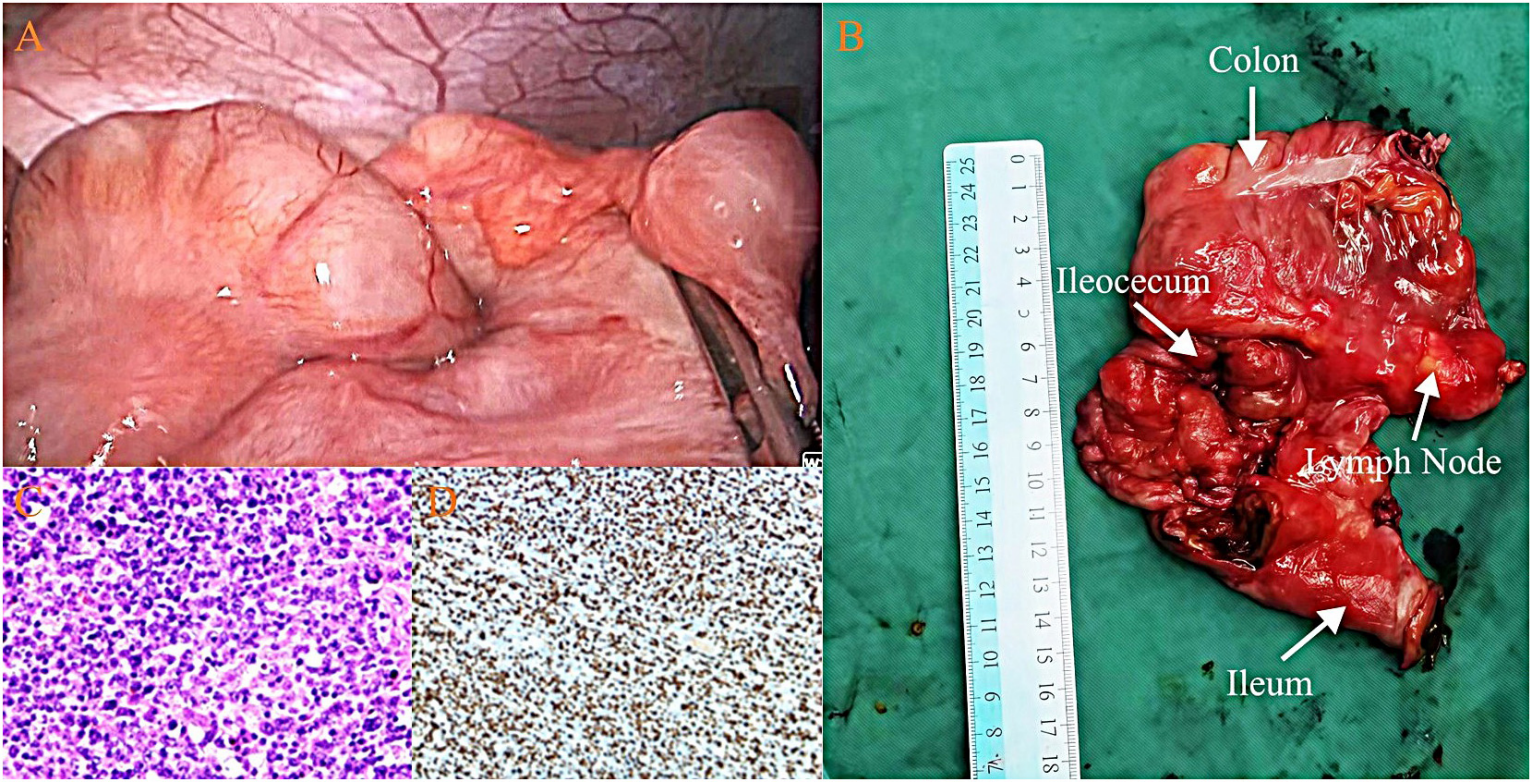
**(A)** Intraoperative view showing the terminal ileum intussuscepted into the ascending colon. **(B)** Pathological specimen after surgical resection. **(C)** Histopathological findings consistent with non-Hodgkin lymphoma. **(D)** Immunohistochemical results supporting the diagnosis of diffuse large B-cell lymphoma (DLBCL), non-germinal center B-cell-like (Non-GCB) subtype.

Pathological examination suggested non-Hodgkin lymphoma. Necrosis with fibrous tissue encapsulation was observed on the mesenteric surface of the ileocecal region. Tumor involvement was identified in one of eleven lymph nodes (1/11). Immunohistochemistry showed that the tumor cells were positive for CD20, CD19, CD79a, and Bcl-2, while negative for CD3, CD10, Bcl-6, and cyclin D1. Ki-67 demonstrated a high proliferation index (90%). The final diagnosis was diffuse large B-cell lymphoma (DLBCL), non-germinal center B-cell-like (Non-GCB) subtype (as shown in Figure 2).

**FIGURE 3:**
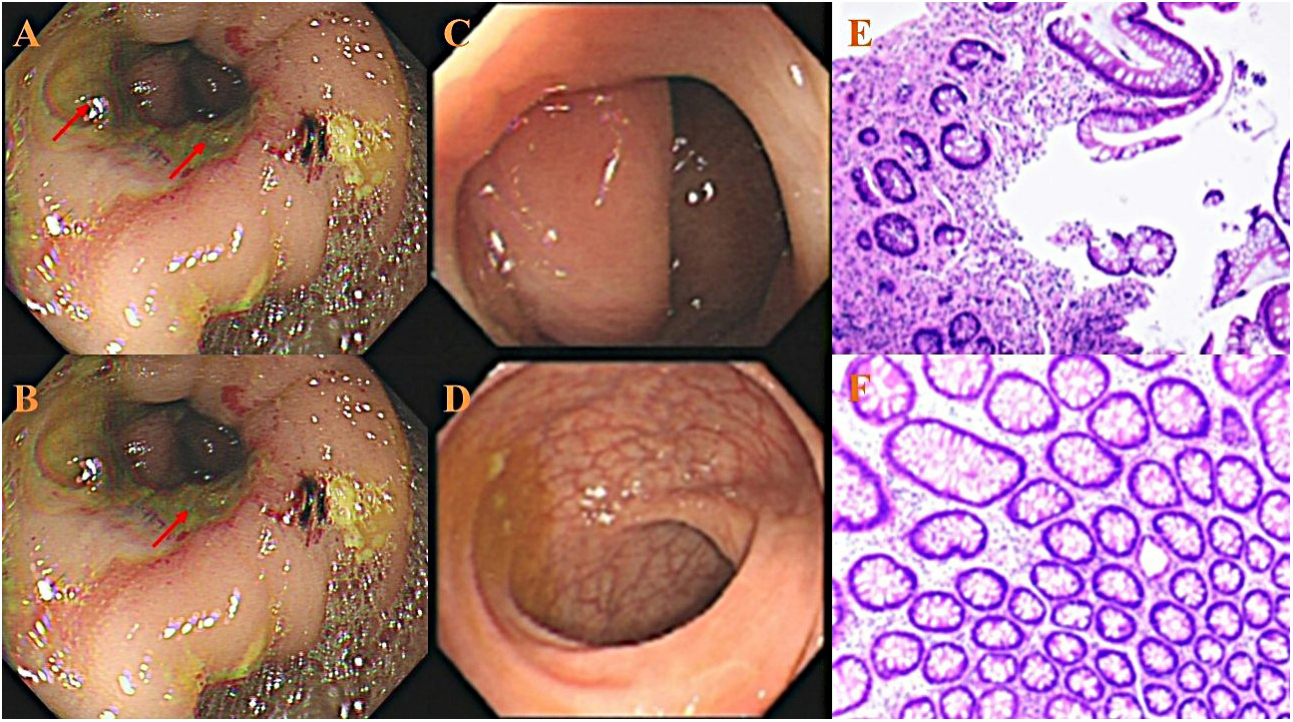
**(A,B)** Colonoscopic images of the small intestine anastomosis site. The arrows indicate extensive ulcers at the anastomosis. **(C,D)** Show the sigmoid colon and rectum, respectively. The mucosa is smooth, with clearly visible vascular patterns. **(E,F)** Pathological biopsy results from the anastomotic ulcers, consistent with the presentation of intestinal ulcers. No tumor cells were identified.

**FIGURE 4:**
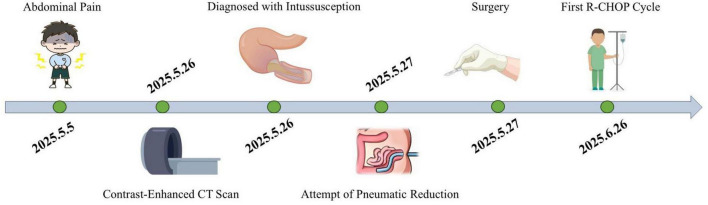
Flowchart of the patient’s clinical course from initial symptom onset to hospital presentation.

The patient’s postoperative diet and bowel function returned to normal. The abdominal drainage tube was removed on the ninth day after surgery. One month postoperatively, the patient was transferred to the hematology department and received treatment with the R-CHOP regimen. Follow-up contrast-enhanced abdominal CT scans performed at 2 and 3 months showed no abnormal densities or enhancement signals. Figure 3 shows a colonoscopy from the 4-month postoperative evaluation, which revealed no abnormal masses in the bowel. (The diagnostic and therapeutic pathway is depicted in the flowchart in Figure 4.)

## Discussion

The clinical symptoms of intussusception were first described by Barbette in 1674 (11). Its classic presentation is characterized by a triad of abdominal pain, a palpable abdominal mass, and currant jelly stool, which is particularly prominent in pediatric cases and exhibits high clinical recognition. In contrast, adult intussusception demonstrates markedly non-specific clinical features and often follows a more subacute (24.4%) or chronic (51.2%) course. Abdominal pain is the most frequent symptom (85%–100%), followed by nausea (41%–75%), vomiting (35%–70%), and bowel dysfunction (22.5%–69%). A palpable mass (9.1%–62.5%) and intestinal bleeding (16.4%–27.3%) are less common and show considerable variability across studies (8, 12–15). Due to this non-specificity, adult intussusception is often misdiagnosed by clinicians as acute appendicitis, chronic enteritis, irritable bowel syndrome, or intestinal obstruction.

Unlike pediatric cases, which are mostly idiopathic, 75%–90% of adult intussusception cases have an identifiable pathological lead point (16). Intussusception occurs when a segment of the intestine becomes fixed due to dyscoordinated peristalsis or an intraluminal obstruction. This leads to the proximal bowel being propelled forward during peristalsis and telescoping into the adjacent distal segment (17). The fixed anatomical or pathological structure that triggers and anchors this process is termed the “lead point.” The nature (benign or malignant) and type of these lead points exhibit distinct distribution patterns across different anatomical subtypes.

According to the location of the insertion point and the sheath, intussusception is mainly divided into: ➀ Ileocolic type: Accounts for approximately 50%–60% of all cases. With the ileocecal valve acting as the leading point, the terminal ileum intussuscepts into the ascending colon. Some cases may involve concurrent intussusception of the cecum and appendix (18, 19). In infants and young children, this type is often idiopathic and may be associated with lymphoid hyperplasia (20). In adults, however, it strongly indicates the presence of an organic pathological lead point. Common benign lead points include mucosal polyps, lipomas, Meckel’s diverticulum, and intestinal adhesions (21), while malignant lesions—such as leiomyosarcoma, adenocarcinoma, gastrointestinal stromal tumor (GIST), carcinoid tumor, neuroendocrine tumors, lymphoma, and metastatic cancer—may account for up to 30% of cases (22, 23). ➁ Ileo-ileocolic type: This refers to intussusception beginning within the ileum (ileo-ileal component), which then progresses through the ileocecal valve into the colon (24). It is often related to local anatomical anomalies or functional disturbances rather than distinct pathological lesions. ➂ Small bowel type: Involves intussusception within the small intestine (e.g., jejuno-jejunal or ileo-ileal). It is relatively uncommon. ➃ Colo-colic type: Occurs when a segment of the colon invaginates into an adjacent colonic segment. This subtype carries significant clinical concern, as up to 80% of cases are associated with malignancy, most commonly primary colonic adenocarcinoma, followed by lymphoma and metastatic tumors (19, 25). ➄ Multiple synchronous intussusceptions: Refers to the simultaneous presence of two or more independent intussusceptions. This rare presentation is typically associated with systemic conditions such as diffuse intestinal lymphoma, metastatic cancer, or extensive postoperative adhesions.

Gastrointestinal lymphoma is the most common form of extranodal lymphoma, accounting for 30%–40% of all extranodal cases but only 1%–4% of all gastrointestinal malignancies (26). It occurs most frequently in the stomach, followed by the ileocecal region, small intestine, and colon (27, 28). Histopathologically, non-Hodgkin lymphoma (NHL) predominates, with diffuse large B-cell lymphoma (DLBCL) comprising approximately 90% of cases. Other less common subtypes include mucosa-associated lymphoid tissue (MALT) lymphoma, follicular lymphoma (FL), mantle cell lymphoma (MCL), Burkitt lymphoma (BL), enteropathy-associated T-cell lymphoma, and NK/T-cell lymphoma (29, 30). Previous studies have linked gastrointestinal lymphoma to factors such as Helicobacter pylori, human immunodeficiency virus (HIV), celiac disease, Campylobacter jejuni, Epstein-Barr virus (EBV), hepatitis B virus (HBV), human T-lymphotropic virus 1 (HTLV-1), inflammatory bowel disease, and immunosuppression (31).

The pathogenesis of DLBCL involves a complex multistep process. Translocations involving BCL6, BCL2, and MYC are observed in 30%–40%, 20%–40%, and 10% of cases, respectively. The germinal center B-cell-like (GCB) subtype is frequently associated with t(14;18)(q32;q21) (involving BCL2), while the activated B-cell-like (ABC) subtype often exhibits abnormalities in 3q27 (BCL6) and 8q24 (MYC) (32–34). Mutations in CD79A/B lead to chronic active B-cell receptor (BCR) signaling and aberrant activation of the NF-κB pathway, playing a particularly critical role in the ABC subtype (35). Although intestinal involvement is uncommon in DLBCL, it warrants clinical attention. We searched all cases of adult DLBCL with intussusception in PubMed, a total of nine cases. It is worth noting that one of the cases is due to the lobulated shape of the tumor leading to imaging findings of pseudo-intussusception (7), which we summarized in Table 1. In previous case reports, all cases were treated for abdominal pain, including our cases. CT is a common diagnostic method; almost all patients underwent surgery combined with chemotherapy, except for one case where the tumor was too large, with extensive lymph node enlargement and splenomegaly, so the patient did not receive surgical treatment (36).

**TABLE 1:** Retrieve adult diffuse large B-cell lymphoma (DLBCL) cases with intussusception in Pubmed.

References	Gender/age	Chief complaint	Diagnostic tools	Type of intussusception	Treatments	CD20 (status)/subtype	Chemotherapy regimen	Outcome
Albrijawy et al. (7)	M/19	Abdominal pain for 2 weeks	CT, ultrasound	Pseudointussusception	Surgery + chemotherapy	CD20+	R-CHOP	CR
Wagh et al. (8)	M/50	Abdominal pain for 2 months	CT, X-ray	Ileocolic type	Surgery + chemotherapy	CD20+/GCB	R-CHOP	CR
Zhang et al. (36)	M/49	Abdominal pain and weight loss for 1 month	CT, PET/CT	Ileocolic type	Chemotherapy	CD20+/Non-GCB	R-CHOP, R-Hyper CVAD (A), R2-GDP	CR
Mathews et al. (37)	M/71	Abdominal pain, nausea, and vomiting for 2 weeks	CT	Ileocolic type	Surgery + chemotherapy	Non-GCB	R-CHOP, clinical trial	Survived for 6 months
Liu et al. (38)	M/84	Abdominal pain, difficulty breathing, fatigue, and progressive generalized edema for 1 month	CT	Small bowel type	Surgery + chemotherapy	NM	R-CHOP	Died 4 months postoperatively
Louis-Jean et al. (39)	M/46	Abdominal pain for 1 month	CT	Ileocolic type	Surgery + chemotherapy	CD20+	R-CHOP	CR
Jin et al. (40)	F/73	Abdominal pain for 1 month	CT	Ileocolic type	Surgery + chemotherapy	CD20+/non-GCB	R-CHOP	Died due to COVID-19
Singh and Mane (41)	F/40	Abdominal pain and vomiting for 1 month	CT, ultrasound	Small bowel type	Surgery + chemotherapy	CD20+/GCB	R-CHOP	PR
Chang et al. (42)	F/71	Abdominal pain and weight loss for 2 months.	CT, X-ray, colonoscopy	Ileocolic type	Surgery + chemotherapy	NM	R-CHOP	CR

CR, complete remission; CT, computed tomography; F, female; M, male; GCB, germinal center B-cell-like; Non-GCB, non-germinal center B-cell-like; NM, not mentioned; PR, partial response.

Diagnosing adult intussusception based solely on clinical symptoms is challenging and requires ancillary investigations. Abdominal computed tomography (CT), with the highest sensitivity, is considered the gold standard for diagnosis (8). It can identify up to 77.8% of cases (43) and clearly demonstrate characteristic features such as the “target” or a “donut sign” soft tissue mass. In tumor-induced intussusception, CT is effective in detecting the lead point mass, assessing its size, morphology, density, and enhancement pattern. Interestingly, the patient we reported showed no abnormal mass or enhancement on CT, with only pure intussusception signs observed. Our interpretation suggests that DLBCL typically exhibits infiltrative growth, spreading longitudinally along the intestinal wall rather than forming an exophytic mass protruding into the lumen. This growth pattern may result in tumor density being similar to that of the surrounding intestinal wall, leading to the absence of significant mass effect or abnormal enhancement on CT. This is particularly evident when the tumor is predominantly located in the submucosal or serosal layer, where it can be easily masked by overlapping soft tissue shadows caused by bowel wall edema or intussusception. Furthermore, the lack of abnormal enhancement on both non-contrast and contrast-enhanced CT in this case may be attributed to tumor necrosis (as indicated by pathological findings of necrosis on the mesenteric aspect) and perfusion characteristics associated with high cellular density. This also highlights certain limitations of CT in determining the nature of the lead points (44).

Ultrasound holds diagnostic value due to its convenience, non-invasiveness, and lack of radiation, making it particularly suitable for radiation-sensitive populations such as children and young adults. Although its accuracy is operator-dependent and may be limited by bowel gas, it remains an important tool for diagnosing intussusception in both children and adults. Abdominal X-ray may suggest intestinal obstruction but has limited diagnostic utility. Magnetic resonance imaging (MRI) is reserved for cases with inconclusive CT or atypical ultrasound findings suggestive of a pathological lead point (8). Other diagnostic modalities include diagnostic laparoscopy, gastrointestinal endoscopy with biopsy, enteroscopy, scintigraphy, angiography, FDG-PET/CT, and tumor markers, each offering certain diagnostic value (38, 45, 46). Although laboratory tests contribute little to the direct diagnosis of intussusception, they are important for evaluating the patient’s general condition and disease severity.

Treatment strategies for adult intussusception are based on the consensus that it is often secondary to an underlying pathology; therefore, surgical resection is the primary approach. Previous studies support radical resection as the treatment of choice, particularly when malignancy is suspected or signs of intestinal ischemia are present. Laparoscopic-assisted resection has gained popularity as a safe, feasible, minimally invasive alternative to open surgery, offering both diagnostic and therapeutic benefits. The question of whether to reduce the intussusception before resection or proceed directly to excision is being scrutinized more carefully. Reduction may be considered in cases where imaging strongly suggests a benign lead point or when reducing long-segment involvement could preserve significant bowel function (47). However, reduction should be avoided when malignancy is highly suspected, as even gentle traction may cause bowel perforation and increase the risk of peritoneal seeding (48, 49). In such scenarios, en bloc resection is mandatory. Even when the nature of the lead point is uncertain, direct resection without attempted reduction is recommended (50). Hydrostatic or pneumatic reduction has very limited applicability in adults and should only be attempted in highly selected cases with confirmed benign etiology, no signs of ischemia or peritonitis, short-segment involvement, and only in settings where emergency surgery is immediately available.

Surgical resection combined with adjuvant chemotherapy significantly improves overall survival (OS) in gastrointestinal DLBCL (51), whereas radiotherapy does not provide a clear benefit (52). The Chinese Society of Clinical Oncology (CSCO) guidelines recommend six cycles of R-CHOP (rituximab, cyclophosphamide, doxorubicin, vincristine, and prednisone) as the standard regimen for young patients with favorable-prognosis DLBCL, with a cure rate exceeding 60% (53). The addition of rituximab should be determined based on CD20 expression status.

Although our initial management appeared to deviate from these recommendations due to limited experience with this condition, no missed diagnosis, misdiagnosis, or surgery-related complications occurred. The case of DLBCL with intussusception we reported offers new clinical insights into diagnostic challenges, age distribution, and treatment decisions compared with previous cases summarized in Table 1. While published cases typically identified tumor masses as lead points through CT imaging, our case showed only the “target” of intussusception on non-contrast and contrast-enhanced CT without detectable masses or abnormal enhancement. This radiologic “false-negative” finding underscores the diagnostic complexity of DLBCL, which may present with infiltrative growth rather than forming distinct space-occupying lesions. It highlights the critical need for clinicians to maintain suspicion for malignancies like lymphoma in adult intussusception—particularly in young patients with subacute or chronic presentations—even when advanced imaging shows no definitive tumor evidence. As shown in Table 1, most previously reported cases occurred in patients over 40 years old, with only one 19-year-old exception. Our 23-year-old patient suggests that the proportion of malignancies (especially lymphomas) may be underestimated in young adults with intussusception, though the condition itself is rare in this population. This necessitates broadening differential diagnoses and avoiding age-based assumptions of benign etiology to prevent diagnostic delays. The decision to attempt pneumatic reduction, while contrary to conventional recommendations, provided valuable clinical reflection. Our outcome ultimately reinforces the consensus in literature: surgical exploration remains the cornerstone of diagnosis and treatment for adult intussusception with unknown etiology, whereas enema reduction should be considered a highly selective and cautious exception.

During the surgery, we initially attempted laparoscopic and open manual reduction. Although resection was ultimately performed due to failed reduction and concerns about bowel injury, the decision-making process reflected our strong commitment to preserving intestinal function in this young patient. We believe that for selected young patients, a cautious attempt at reduction—with the goal of maximizing preservation of healthy bowel while ensuring complete tumor resection—may represent a reasonable individualized approach. This, however, requires rigorous intraoperative risk-benefit assessment and readiness to proceed decisively with resection if needed.

## Conclusion

In summary, adult intussusception is a rare clinical entity that often masks an underlying malignancy, with diffuse large B-cell lymphoma (DLBCL) being one of the significant etiologies. Its non-specific clinical presentation frequently leads to misdiagnosis. Abdominal CT serves as the primary diagnostic tool, though definitive diagnosis relies on pathological and immunohistochemical examination. The recommended treatment strategy involves radical surgical resection combined with systemic chemotherapy, such as the R-CHOP regimen. Clinicians should maintain a high index of suspicion for adult intussusception, as early recognition, accurate diagnosis, and standardized multimodal therapy can significantly improve patient outcomes.

## Data Availability

The original contributions presented in this study are included in this article/supplementary material, further inquiries can be directed to the corresponding author.
